# From Functional Ingredients to Functional Foods: Focus on Brassicales Plant Species and Glucosinolates

**DOI:** 10.3390/foods15030537

**Published:** 2026-02-03

**Authors:** Eleonora Pagnotta, Roberto Matteo, Luisa Ugolini

**Affiliations:** CREA—Council for Agricultural Research and Economics, Research Centre for Cereal and Industrial Crops, Via di Corticella 133, 40128 Bologna, Italy; roberto.matteo22@outlook.com (R.M.); luisa.ugolini@crea.gov.it (L.U.)

**Keywords:** glucosinolates, phytochemicals, food application, chemoprevention, value-added products, health benefits

## Abstract

The concept of functional nutrition has garnered mounting attention, primarily due to growing evidence that specific dietary components have the capacity to provide health benefits that extend beyond the mere supply of basic nutrients. In this context, glucosinolate-rich species of the Brassicales order are of importance as a source of bioactive compounds, which exhibit antioxidant, anti-inflammatory, and chemoprotective properties. The review identifies which Brassicales species may be considered as functional foods or functional ingredients. It does so by starting from their glucosinolate profile, summarizing their potential applications in disease prevention, and highlighting current strategies aimed at enhancing glucosinolate levels through agronomic practices and processing approaches. The potential applications of the main species of the Brassicales order in the prevention of cardiovascular, obesity-related and degenerative diseases, as well as in the development of functional foods, are highlighted. These species are considered both as ready-to-use functional foods and as functional ingredients that can be obtained through extraction or fermentation processes, including the valorization of agricultural waste.

## 1. Introduction

The notion of functional nutrition has garnered significant attention within the scientific community and with consumers. This interest has been propelled by mounting evidence suggesting that specific dietary components possess the capacity to impart health benefits that extend beyond basic sustenance and energy provision. A functional ingredient may be defined as a bioactive compound that provides health-promoting effects beyond basic nutritional requirements. A functional food is an ordinary edible product consumed as part of the regular diet with the aim of delivering physiological benefits or reducing the risk of chronic diseases. The primary approaches adopted to produce processed functional foods include enrichment, fortification, and biofortification. These methods contrast with natural functional foods, which are constituted by raw fruits, vegetables, and grains and are abundant in essential nutrients and phytochemicals [1]. The term “enrichment” is typically employed to denote the process of supplementing a food source with selected micronutrients that are naturally present in the food, albeit in quantities that are insufficient. Fortification, on the other hand, involves the addition of essential micronutrients to a food that is inherently deficient in a specific nutrient. This process is particularly relevant for individuals who are deficient in micronutrients due to malnutrition, a condition that is prevalent among certain populations in developed countries [2]. The term “biofortification,” meanwhile, refers to the agronomic or genetic practices of enriching food crops with specific bioactive nutrients [3]. In the broad category of natural functional foods, broccoli, *Brassica oleracea* var. *italica* Plenck, is regarded as an example of a food with verified anticancer, antioxidant, and anti-inflammatory properties [4].

Broccoli belongs to the brassica vegetable family and is a good source of bioactive molecules and micronutrients, including vitamin C, β-carotene, calcium, and fibre [4]. However, these vegetables also retain glucosinolates (GSLs) in their composition. GSLs are anionic secondary metabolites that contain at least two sulfur atoms included in an *O*-sulfated (*Z*)-thiohydroximate function and are characteristic of all members of the plant order Brassicales [5]. GSLs themselves are not bioactive, at least at the concentration found in commonly eaten vegetables, but their typical hydrolysis products at physiological conditions, the isothiocyanates (ITCs), can be highly bioactive [6]. Glucoraphanin is the predominant GSL in broccoli. Other notable GSLs include glucoiberin, glucoerucin, and indolic GSL, with content varying by cultivar, developmental stage, and storage period. The ITC metabolites of these GSLs, particularly sulforaphane (SF) and indole-3-carbinol, have been associated with anti-inflammatory, antioxidant, and chemoprotective effects [4]. The incorporation of GSL-rich foods into one’s daily diet seems to be linked to a reduced incidence of chronic diseases, a reduced risk of various cancers, the prevention of degenerative diseases like Alzheimer’s, and a lower incidence of cardiovascular diseases [6,7].

The interest in the effects of this molecular system on human health is confirmed by numerous reviews published in the recent literature. These reviews have addressed and collected the available experimental data on the bioavailability, metabolism, intervention studies [8], pharmacological properties, and toxicological concerns, mainly related to the Brassicaceae plant family and brassica seed utilization [9,10]. The *Brassica oleracea* species and its varieties appear as those mainly described, reflecting their widespread use in human nutrition. However, the health impacts of other species have also been recently summarized, including *Armoracia rusticana* [11] which also belongs to the Brassicaceae family, but also *Moringa oleifera* [12,13,14] from the Moringaceae family, and *Capparis spinosa* from the Capparaceae family [15]. Horseradish roots are particularly rich in sinigrin, whose isothiocyanate is primarily responsible for its pungent taste; a recent review [12] provided an overview of the moringa species, their countries of origin, and their known therapeutic uses. This was accompanied by a comprehensive overview of the activities of the most studied species in recent years, *M. oleifera* Lam. The organs of this species have been extensively characterized phytochemically in recent years, revealing exclusively the presence of aromatic GSLs, with glucomoringin, 4-(α-l-rhamnopyranosyloxy)benzyl GSL, predominating in moringa seeds and its acetylated isomer III, 4′-O-acetyl-4-(α-l-rhamnopyranosyloxy)benzyl GSL, generally more concentrated in the leaves [14]. Methyl GSL, glucocapparin, undergoes hydrolysis to form methyl isothiocyanate which is mainly responsible for the pungent taste of its flower buds [16].

According to the Food and Agriculture Organization of the United Nations, in 2022, the global production of cruciferous crops reached a total of 72,604 kilotons with a 6.53% increase compared to the data of previous ten years [17], while if we restrict on global production of broccoli and cauliflower, this increase was about 48% in the period from 2003 to 2023 [18]. It was estimated that the edible part of broccoli is limited to a 15% of florets, while the remaining parts are discarded during harvesting, householding, and commercial processing. When the entire Brassicales order is taken into consideration, i.e., all species containing GSLs, the possibility of considering other types of waste increases considerably among all edible species. The order Brassicales is characterized by a high level of diversity, encompassing a wide range of species, traits, and environmental adaptations. There are approximately 4700 species which are divided into 18 families. This diversity is distributed over a considerable geographical area and includes several known crops such as canola, caper, broccoli, kale, papaya, rapeseed, and saltwort [19]. It is important to note that not all plants in the Brassicales order are edible, but many of them are cultivated crops and can be used as ingredients for foods, functional foods, or supplements.

The present review has thus been conceived with the objective of identifying which species of the Brassicales order are currently considered as functional foods or ingredients based on their GSL content. A particular focus has been placed on agronomic and/or processing strategies for biofortification, as well as on the relationship between diseases and nutraceutical applications. The review highlights a growing interest in cultivating microgreens and using mild technologies, such as fermentation, to exploit the health properties of not only Brassicaceae, but also species belonging to other plant families that are classified within the Brassicales order.

## 2. Methods

A comprehensive search was performed on the electronic database Scopus including articles published from 2009 to 20 August 2025. The study incorporated a comprehensive range of research designs, encompassing in vitro cell models, in vivo animal models, and clinical trials involving human subjects. Excluded from the analysis were review articles and books, as well as book chapters. The search terms employed in the present study included the following keywords: “glucosinolates”, “functional”, “food”, “Brassicaceae”, and “Brassicales”. During the research period, which occurred from 1 July 2025 to 20 August 2025, a total of 210 articles were initially identified. These were then subjected to further screening, whereby articles that failed to clearly report the species considered or did not provide sufficient information on plant growth, extraction processes, or the use of materials in the case of commercial products, were excluded. Finally, articles focusing solely on the phytochemical characterization of materials or on purified GSLs, and which did not relate to the design of functional foods, were also excluded from the final selection.

## 3. Raw Vegetables from Brassicales Order as Unprocessed Functional Foods

The first evaluation was conducted on articles that considered plant tissues from mature plants, microgreens, or sprouts of species belonging to the Brassicales order as potential functional foods or as possible ingredients in foods with clear nutraceutical applications. A total of 55 articles were considered. Table 1 summarizes the main agronomic growth strategies, where available, along with the processing approaches adopted prior to the evaluation of their properties and the biochemical outcomes, primarily in terms of the enhancement of bioactive compounds content and bioavailability, antioxidant activity, angiotensin converting enzyme (ACE) and α-amylase inhibitory activities, suppression of lipid accumulation, and nutraceutical applications. The period under consideration was limited to 2020–2025, with the intention of emphasizing the most recent trends in research within this field.

**Figure 1:**
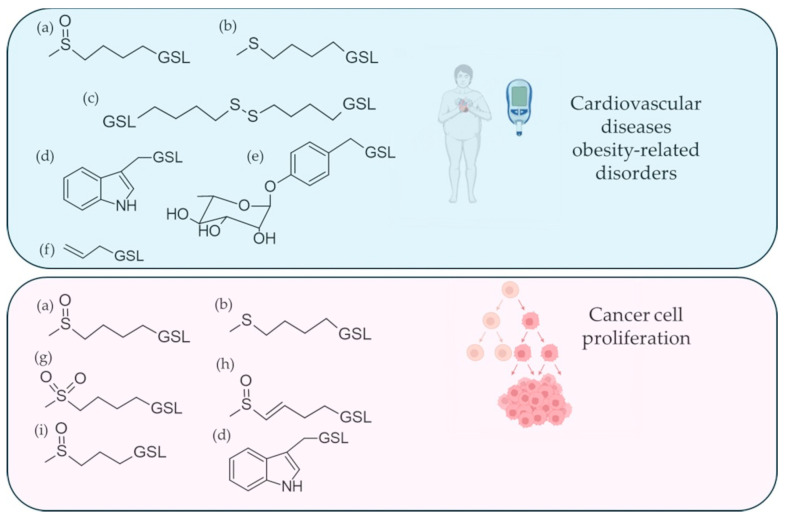
Examples of glucosinolates (GSL), which are primarily found in species that are considered as functional foods for the prevention of cardiovascular disease, obesity, and cancerous degeneration. (a) (*R*_S_)-4-(Methylsulfinyl)butyl GSL (Glucoraphanin); (b) 4-(Methylthio)butyl GSL (Glucoerucin); (c) Dimeric 4-mercaptobutyl GSL; (d) Indol-3-ylmethyl GSL (Glucobrassicin); (e) 4-(*α*-l-Rhamnopyranosyloxy)benzyl GSL (Glucomoringin); (f) Prop-2-enyl GSL or Allyl GSL (Sinigrin); (g) 4-(Methylsulfonyl)butyl GSL (Glucoerysolin); (h) (*R*_S_, 3*E*)-4-(Methylsulfinyl)but-3-enyl GSL (Glucoraphenin); (i) (*R*_S_)-3-(Methylsulfinyl)propyl GSL (Glucoiberin). The figure was partially created in Biorender by EP (2026), https://BioRender.com (accessed on 25 January 2026).

In recent years, research interest in functional food production has shifted decisively toward the cultivation of microgreens, with *Brassica oleracea* and its varieties being the most represented species (43% of the studies considered) and *Raphanus sativus* the second species of interest. Research in this area is characterized by extensive experimentation in the agronomic field, with the aim of identifying the optimal growing conditions for maximizing the fortification of the nutraceutical properties of the products. Growth with different photoperiods, or the use of LED lights at different wavelengths [39], as well as on different substrates [51] supplemented with various enrichments, or treatments with Zn [46], potassium, or CaCl_2_ [43], have been explored to verify the qualitative and quantitative change in bioactive molecules, the maintenance of properties over time, and the reduction in unwanted GSL such as progoitrin. In contrast to the growing prevalence of research on microgreens in recent years, studies focusing on sprouts have declined. The only species classified outside the Brassicaceae family as a functional food, in both mature leaves and sprouts, is *Moringa oleifera*. *Moringa oleifera* sprouts offer a GSL composition that is not significantly different from that of seeds, which have a substantial fibre content, and an interesting protein content. In addition, *Moringa oleifera* sprouts are enriched with γ-aminobutyric acid, a well-known neurotransmitter and blood pressure regulator, and linked to the prevention of diabetes and diuresis regulation [59]. Regarding the examination of nutraceutical applications, Table 2 provides a comprehensive overview of the diseases primarily considered in relation to the chemopreventive properties of the various species, while Figure 1 highlights the GSL structures most commonly identified in species normally used as food that may be effective in protecting the organism, especially from cardiovascular diseases and obesity-related disorders, but also in protecting against malignant degenerative processes.

The analysis also encompassed scientific articles published prior to 2020, thereby incorporating species not only belonging to the Brassicaceae and Moringaceae families, but also *Maerua subcordata*, which belongs to the Capparaceae family. The latter is distinguished by a high content of glucocapparin and stachydrine in its tissues [61]. This medicinal plant, native to East Africa, has demonstrated safety in in vitro tests conducted on its seeds, fruits, and roots, indicating no observed signs of toxicity [63].

## 4. Fermented Foods or Fermented Ingredients from Brassicales Order as New Functional Options for Food Industry

**Figure 2:**
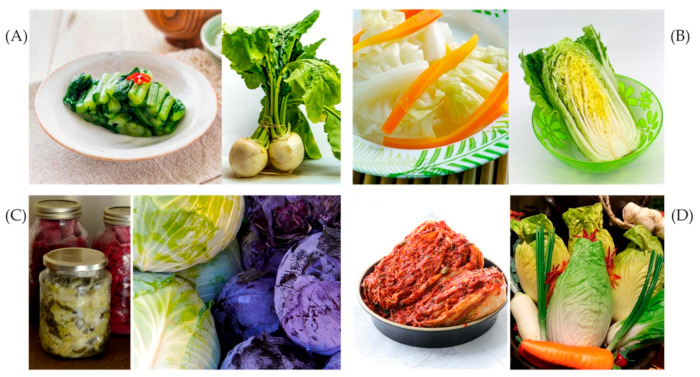
Traditional fermented dishes made from plants of the Brassicales order and species derived from them: (**A**) Japanese nozawana from *Brassica rapa* L. leaves; (**B**) Chinese pao cai from *Brassica rapa* subsp. *pekinensis* (Lour) Hanelt; (**C**) sauerkraut from *Brassica oleracea* var. *capitata* L.; (**D**) Korean kimchi from *Brassica rapa* subsp. *pekinensis* (Lour) Hanelt and *Rapahnus sativus* L.

While interest in various species of Brassicaceae as ready-to-use functional foods has increased in recent years—particularly with respect to microgreens—there has also been a growing focus on fermented foods among processed ones. Fermented foods encompass a wide variety of products transformed by different strains of microorganisms, which induce biochemical changes, modify food taste and storage life, and improve functional properties [64]. Several fermented products are derived from the Brassicaceae family. The range of products includes sauerkraut, which is popular in Europe and the USA; kimchi, a traditional Korean dish made from fermented napa cabbage, radish, red chillies, garlic, and fish; pao cai, considered a symbol of southwestern Chinese culture and made from fermented napa cabbage, like kimchi; and Japanese products such as sunki and nozawana, which are produced in Japan from turnip leaves [65] (Figure 2).

At least ten different species belonging to the Brassicales order have been subjects of scientific research in recent years. These species encompass members representative of the Brassicaceae family, as well as those belonging to the Caricaceae and Moringaceae families. These fermented foods display a rich diversity of microorganisms, primarily lactobacilli, which are responsible for the production of lactic acid, as well as an increased concentration of vitamins, GSL hydrolysis products, short chain fatty acids, polyphenols, and other bioactive compounds. This considerably expands the range of applications of these foods, particularly in relation to disorders of the intestinal tract, compared to non-fermented vegetables. Table 3 provides an overview of the diseases and fermentable Brassicales species that have demonstrated chemopreventive effects. These diseases include inflammatory diseases, particularly those affecting the intestine, as well as the airways, the control of pathogenic bacteria, and the reduction in possible antinutrients in plant matrices, with advantages in terms of product safety.

Fermented products have been demonstrated to be an effective solution for obtaining products and juices that are particularly rich in ITCs and ascorbigen after a relatively brief period of spontaneous fermentation, as evidenced in the case of white cabbage sauerkraut [70], or the same species fermented with myrosinase-positive bacteria, a process which has been shown to result in increased concentrations of sulforaphane and iberin [75]. The most widely studied combination of lactobacilli for enhancing the bioavailability of GSL hydrolysis products in broccoli is a mixture of *Lactobacillus plantarum* and *Leuconostoc mesenteroides* [76,77]. The same lactobacilli, together with *Pediococcus acidilactici*, have also been used on moringa leaves, achieving good results in terms of increasing antioxidant capacity and improving the phenolic profile [78]. Finally, the potential application of fermentation technologies to reduce the presence of anti-nutritional compounds, such as phytates, tannins, and oxalates, in blanched Brassica oleracea sprouts inoculated with *Lactobacillus plantarum*, represents a highly intriguing avenue for further research [74]. Table A1 in Appendix A provides a comprehensive overview of fermented foods derived from species belonging to the Brassicales order that have recently been recognized for their potential health benefits. These foods, and the techniques used to produce them, are also detailed, when available. Fermentation technology is being viewed with increasing favour as a means of reusing waste materials from diverse origins to obtain new ingredients that offer enhanced health benefits. For instance, the substantial volume of meal produced on an annual basis from the de-oiling of double-low rapeseed meal for human consumption could be utilized for this purpose [67]. Additionally, portions of broccoli that are typically discarded as unsuitable for the market could also be effectively reused [79].

## 5. By-Products from Brassicales Order Species as New Functional Ingredients

When considering the different types of waste produced by the most widely used commercial plant species belonging to the Brassicales order in the food industry, the potential for developing new plant-based ingredients—and for transforming waste into products with increased nutraceutical value—expands considerably. The recent literature has predominantly focused on waste derived from *Brassica oleracea* var. *italica* Plenck starting from seed lots rejected due to poor grain quality, low germination rate, or other yield-related parameters [80] and extending to broccoli deemed commercially unsellable because of post-harvest issues related to abiotic stresses, mainly wounding [81]. However, the main emphasis has been on broccoli leaves and stems [82,83,84,85,86,87]. The unused leaves of the species *Raphanus sativus* [88], *Brassica oleracea* var. *capitata* [86], and *Brassica rapa* [89] were also evaluated. Finally, due to their GSL and residual ITC content, as well as their potential applications in the food and beverage sector, the stems and roots of papaya have recently been considered too [90]. Defatted meals from Brassica seeds that have been selected based on their GSL profile and content have also been considered interesting as ingredients in functional foods, as in the case of *Eruca sativa* meals with a high glucoerucin content [91,92,93,94]. Table 4 provides an update on each by-product of species belonging to the Brassicales order that have been considered in recent years for nutraceutical applications.

## 6. Main Glucosinolates and Their Hydrolysis Products Identifiable in Species of the Brassicales Order of Interest for Functional Nutrition

The species belonging to the Brassicales order offer a plethora of possibilities for the intake of nutrients that are beneficial to human health, as evidenced by the wide variety of GSLs they contain. The application of agronomic techniques and the study of post-harvest processes are broadening the range of possibilities in this field. Concurrently, these techniques can assist in the reduction in GSLs, which are regarded as anti-nutritional, such as progoitrin. To date, this is one of the primary GSLs to be monitored to maintain low concentrations, particularly in the context of enrichment, fortification, and biofortification processes [95]. As illustrated in Table 5, a thorough investigation of the species examined in this review is provided, along with a detailed analysis of the predominant GSLs they contain. In order to establish the profile of the main GSLs, the focus was on the mature organs that are typically utilized in food applications. However, certain lesser-known species or those for which the seed is generally employed, or those that are primarily used as microgreens, were included and highlighted in brackets in the first column.

The only species not included in Table 5 is *Brassica napus*, which is considered in this research only as defatted meal. This is because *B. napus* is particularly rich in progoitrin and has already been subjected to breeding programs aimed at reducing its overall GSL content. Furthermore, it is considered only as an application of fermentation processes, precisely with the aim of further reducing its content [67]. It has been observed that other species, including *Brassica oleracea* var. *gemmifera* DC. and *Brassica rapa* subsp. *chinensis* (L.) Hanelt, despite exhibiting an intriguing GSL profile, have been found to contain elevated levels of progoitrin. However, research in the field of agronomy and fermentation technologies suggests that there may be ways to modulate the content of this undesirable GSL [44,57,67]. Among the GSLs highlighted in Table 5, in addition to progoitrin, another GSL has been identified as a potential safety concern in plants. This is sinalbine, the primary GSL present in *Sinapis alba* seeds. The hydrolysis of this GSL in an aqueous environment produces the intermediate para-hydroxybenzyl alcohol, which can dimerize to an isomer of bisphenol F. Although there is a paucity of comprehensive studies on the toxicity of this molecule, its similarity to bisphenol A suggests caution, particularly regarding its effects on the endocrine system [111]. As specified in Table 5, the main species and GSLs that characterize the plant organs primarily used as food are listed; in the case of *Sinapis alba*, these organs are the seeds. In García-Pérez et al. recent study [40], microgreens from various Brassicaceae, including *Sinapis alba*, were characterized following biostimulant treatments with commercial vermicompost at multiple doses. The lowest doses resulted in the elicitation of GSLs in the microgreens, which exhibited a complex profile of short- and long-chain aliphatic, aromatic, and indole GSLs. Conversely, the higher doses led to the suppression of GSL synthesis in comparison to the untreated controls. These profiles will likely require verification through the use of standards and other analytical techniques. However, the study underscores the potential of seed priming treatments with vermicompost as a promising strategy for enhancing the accumulation of aliphatic GSLs, in addition to boosting the antioxidant and neuroprotective properties of Brassicaceae. Returning to GSLs with beneficial effects on human health, glucoraphanin, the precursor of sulforaphane, is found in broccoli, but also in various species in the Eruca genus, which are typically consumed raw. It is also present in *Brassica oleracea* var. *viridis* L., *Raphanus sativus* L., and in microgreens of *Brassica rapa* subsp. *nipposinica* (L.H. Bailey) and *Brassica rapa* L. The latter also contain significant amounts of glucoerucin, a reduced analogue of glucoraphanin, which has recently been studied for its protective properties on the cardiovascular system [112], and it is particularly prevalent in *Brassica oleracea* var. *gongylodes* L. [44]. Beyond the confines of the Brassicaceae family, *Moringa oleifera* Lam and *Carica papaya* L. stand out as noteworthy species. The former species is remarkable for its distinctive GSL profile and the favourable effects documented in scientific literature [14], while the latter merits particular attention for the reuse of its waste products, particularly stems, roots, and peel [90], which contain high concentrations of glucotropeolin and may therefore warrant further evaluation.

## 7. Conclusions

The present review consolidates current evidence supporting the Brassicales order as a rich and versatile source of functional foods and ingredients, primarily due to the presence of GSL and their hydrolysis products. Recent studies highlight the growing relevance of raw matrices such as microgreens, which combine high concentrations of bioactive compounds with flexible agronomic strategies for targeted biofortification. In parallel, food processing approaches—particularly fermentation—have proven effective in enhancing the bioavailability of ITCs, reducing antinutritional factors, and expanding the range of health-promoting applications associated with Brassicales-derived products. Moreover, the valorization of by-products from both primary production and food processing represents a promising strategy to align functional food development with sustainability and circular economy principles. Despite the substantial progress achieved at the experimental level, the translation of these findings into market-ready foods remains limited by the scarcity of standardized processing protocols and systematic safety control strategies that are essential for the safe use of waste in food applications. The establishment of clear international regulations on raw material production methods is imperative, necessitating the implementation of good agricultural practices. Furthermore, postharvest handling should minimize microbial and fungal contamination by ensuring timely transport and refrigeration or accelerated drying methodologies, accompanied by minimal energy consumption. Future research should therefore focus on integrating technological optimization with clinical validation to support health claims and promote the effective incorporation of Brassicales-based functional foods into the human diet.

## Figures and Tables

**Table 1:** Functional foods derived from Brassicales: species, processing, biochemical outcomes, and health applications.

Kind of Functional Food	Brassicales Order Species	Agronomic Growth Strategy and/or Processing	Nutraceutical Application	Ref.
Raw vegetables	*Diplotaxis tenuifolia* (L.) DC.	*Sorghum bicolor* L. manure was applied prior to sowing, followed by solarization and standard input allowed in organic cultivation. Three non-microbial biostimulants were compared.	Enhancement of bioactive compound synthesis such as glucoerucin or ferulic acid for diverse dietary applications.	[20]
	*Diplotaxis tenuifolia* (L.) DC.	Three commercial hybrids were sown in autumn and received different foliar fertilization treatment during the crop cycle consisting of three harvest cut and re-growth periods (autumn–winter, winter–spring, spring–summer).	Enhancement of bioactive compound synthesis and antioxidant activity for diverse dietary applications.	[21]
	*Diplotaxis tenuifolia* (L.) DC.	A commercial hybrid was sown in autumn and received several treatments (selenium, iodine, and selenium + iodine) during the crop cycle consisting of three harvest cut and re-growth periods (November–December, December–January, January–March).	Treatments increased both yield, antioxidant activity and accumulation of selenium, iodine, potassium, and calcium, useful to prevent human deficiency and to protect against cardiovascular disease.	[22]
	*Diplotaxis muralis* DC.	Grown in soil, leaves were harvested prior to the flowering stage.	Leaves are rich in dietary fibre, essential minerals, amino acids, and fatty acids, with α-linolenic acid being predominant; GSL and flavonols are present and make the plant interesting for the development of functional foods.	[23]
	*Nasturtium officinale* R.Br.	Dried aerial parts were extracted in boiling water.	Watercress extract could be a functional food for hyperthyroidism; however, it may worsen hypothyroidism by increasing both thyroid and body weight in animal mode.	[24]
	*Brassica oleracea* L.	Commercial samples were subjected to freeze-drying or air frying (160° for 10 min) thermal treatments.	Air frying enhanced linolenic acid and phytosterol levels, and maintained ACE inhibitory activity and α-Amylase inhibitory activity: air-frying represents a promising process for food product development.	[25]
Raw vegetables (continued)	*Brassica oleracea* var. *capitata* L.	Fresh red cabbage was chopped into small pieces, blanched for 30 s in boiling water, and rapidly cooled in ice water. Then they were dried in a convective hot air dryer at different temperatures in the range of 50–90 °C.	Functional food ingredient with neuroprotective capacity against Parkinson’s disease. Drying at 50 and 90 °C allows the highest retention of GSL, flavonoid, and antioxidant activity (Orac assay) together with a significant effect on cytotoxicity triggered by α-synuclein accumulation in a cellular model of Parkinson’s disease.	[26]
	*Brassica oleracea* var. *gemmifera* DC.	Commercial sample was subjected to freeze-drying or air frying (160° for 10 min) thermal treatments.	Air frying enhanced linolenic acid and phytosterol levels, and increased both ACE inhibitory activity and α-Amylase inhibitory activity: air-frying represents a promising process for food product development.	[25]
	*Brassica oleracea* var. *italica* Plenck	Grown and harvested in farm, freeze-dried, and powdered heads.	Prevention or treatment of Alzheimer’s disease thank to its enrichment in phenolic and GSL compounds; among these, kaempferol 7-glucoside and glucobrassicin are the main metabolites potentially involved in anticholinesterase activity.	[27]
	*Brassica oleracea* var. *italica* Plenck	Commercial florets were frozen at −20 °C for 15 days and boiled for 10 min or steamed in an oven by steam injection at 99 °C for 13 min.	Steaming preserved GSL in broccoli and promoted plasma bioavailability of ITCs and lipophilic antioxidants such as lutein and β-carotene.	[28]
	*Brassica oleracea* var. *italica* Plenck	Florets were processed at high pressure (3 min treatment at 400 MPa), combined with microwave treatment (460 W for 20 s).	Improvement of sulforaphane production after chewing.	[29]
	*Brassica oleracea* var. *italica* Plenck	Broccoli were processed at different temperatures, pH and in emulsion with oil, before freeze-drying.	Improvement of sulforaphane release and stability in freeze-dried broccoli powders. pH 5 or pH 6 were the optimum conditions for sulforaphane production and an oil enriched in omega-3 fatty acids could help preserve sulforaphane from degradation.	[30]
	*Brassica oleracea* var. *italica* Plenck	Broccoli heads were steamed in pre-heated oven for 5 min, freeze-dried and then mixed in high fat diet, in comparison to a normal diet and a high fat diet enriched with 150 µmol/kg body weight of pure glucoraphanin.	Modulation by dietary broccoli or glucoraphanin of obesity-related disorders in mice: focus on lipid levels, insulin resistance and gut microbiota.	[31]
Raw vegetables (continued)	*Brassica oleracea* var. *viridis* L.	Freeze dried leaves of kale were mixed to a certified laboratory feed to produce pellets with 10, 30, and 60 g/kg kale enriched feed for long term nutrition experiment (90 days) in rats.	Safety assessment of a long-term continuous kale diet. An improvement of antioxidant defences was observed and verified by increased activities of catalase, superoxide dismutase, glutathione reductase and glutathione S-transferase in the liver, as well as enhanced antioxidant potential in the plasma.	[32]
	*Brassica juncea* (L.) Czern	Commercial plants were washed, freeze dried, and extracted in 60% ethanol to obtain a powdered extract standardized in sinigrin (18 mg/g or 50 µmol/g).	Obesity control. The standardized *B. juncea* extract inhibited in vitro lipid accumulation and improved high fat diet induced obesity in animals by suppressing the formation of adipocytes and promoting fatty acid oxidation and thermogenesis related proteins.	[33]
	*Brassica juncea* (L.) Czern	Standardized *B. juncea* extract was produced with 60% ethanol at 70 °C for 3 h and tested in comparison with sinigrin.	Obesity control. Suppression of lipid accumulation and ROS production.	[34]
	*Brassica rapa* subsp. *chinensis* (L.) Hanelt	Screening of 23 accessions of the Choy sum germplasm available at the National Agrobiodiversity Center (RDA-Genebank) of the Rural Development Administration, Jeonju, Republic of Korea for their GSL content.	Identification of varieties rich in aliphatic GSL for breeding purposes. Varieties rich in glucobrassicanapin showed high content of progoitrin which may reduce functional food applications.	[35]
	*Moringa oleifera* Lam.	Dried powder of *M. oleifera* leaves were extracted by sonication in 90% ethanol. The resulting extract was concentrated and lyophilized to produce a dry powder.	Treatment of hyperglycemia and hyperlipidemia. Among GSL and phenolic compounds, the main bioactive components of the extract responsible for its hypoglycemic and hypolipidemic effects were found to be quercetin and kaempferol monoglycoside-based flavonoid glycosides.	[36]
Microgreens (days form seeding to harvest 8–14)	*Diplotaxis tenuifolia* (L.) DC.	Microgreens were grown with reduced potassium K regimes.	Chronic kidney disease.	[37]
	*Eruca vesicaria* subsp. *sativa* (Mill.) Hegi	Microgreens were grown in commercial micro-farm and harvested without roots.	Prediction of nutritional outcome through in vitro simulated digestion and human fermentation ex vivo. *E. sativa* exhibited a high content in flavonoids and stilbenes and a high percentage of bioaccessibility after simulated static gastrointestinal digestion and in vitro large intestine fermentation step for phenolic compounds.	[38]
	*Eruca vesicaria* subsp. *sativa* (Mill.) Hegi	Microgreens were grown in controlled conditions (22 °C temperature, 60% relative air humidity). Seeds were germinated for three days in darkness and then, until harvest time (12 days), were grown under fluorescent or LED lamps with a photoperiod of 16 h light/8 h dark or continuous light.	Continuous LED light (red:green:blue ratio was 50.3:21.1:17.6) may improve antioxidant properties in microgreens.	[39]
	*Brassica oleracea* L.	Microgreens were grown with reduced potassium K regimes.	Chronic kidney disease	[37]
	*Brassica oleracea* L.	Microgreens were grown in commercial micro-farm and harvested without roots.	Prediction of nutritional outcome through in vitro simulated digestion and human fermentation ex vivo. *B. oleracea* exhibited a high percentage of bioaccessibility after simulated static gastrointestinal digestion and in vitro large intestine fermentation step for GSL and ITC.	[38]
	*Brassica oleracea* var. *capitata* L.	Microgreens were cultivated in a vertical growth chamber under controlled light and temperature conditions.	Suppression of GSL and accumulation in polyphenols, and improvement in antioxidant activity large intestine fermentation step for GSL and ITC.	[40]
	*Brassica oleracea* var. *capitata* L.	Microgreens were grown in commercial micro-farm and harvested without roots.	Prediction of nutritional outcome through in vitro simulated digestion and human fermentation ex vivo. *B. oleracea* var. *capitata* exhibited a high percentage of bioaccessibility after simulated static gastrointestinal digestion and in vitro large intestine fermentation step for GSL and ITC.	[38]
	*Brassica oleracea* var. *capitata* L.	14-day young shoots were grown in a greenhouse in sowing boxes, and the same seedlings were then planted in the ground to obtain mature vegetables.	Diet enrichment in vegetable proteins, minerals, and GSL in comparison to mature headed cabbages.	[41]
Microgreens (continued)	*Brassica Oleracea* var. *botrytis* L.	Microgreens were grown in commercial micro-farm and harvested without roots.	Prediction of nutritional outcome through in vitro simulated digestion and human fermentation ex vivo. *B. oleracea* var. *botrytis* exhibited a high percentage of bioaccessibility after simulated static gastrointestinal digestion and in vitro large intestine fermentation step for GSL/ITC, anthocyanins, and stilbenes.	[38]
	*Brassica oleracea* var. *italica* Plenck	Microgreens were grown in commercial microfarm an harvested without roots.	Prediction of nutritional outcome through in vitro simulated digestion and human fermentation ex vivo. *B. oleracea* var. *italica* exhibited a high percentage of bioaccessibility after simulated static gastrointestinal digestion and in vitro large intestine fermentation step for GSL/ITC.	[38]
	*Brassica oleracea* var. *italica* Plenck	Microgreens were grown in controlled conditions (22 °C temperature, 60% relative air humidity). Seeds were germinated for three days in darkness and then, until harvest (12 days), were grown under fluorescent or LED lamps with a photoperiod of 16 h light/8 h dark or continuous light.	Continuous LED light (red:green:blue ratio was 50.3:21.1:17.6) may improve antioxidant properties in microgreens.	[39]
	*Brassica oleracea* var. *italica* Plenck	Broccoli seeds were germinated in the dark for four days. The seedlings were then exposed to light (12 h/12 h light/dark) and sprayed once a day with H_2_O for six days. Finally, microgreens were harvested 10 days after sowing, roots were removed, and microgreen juice was produced, freeze and stored at −80 °C.	Prevention of obesity via gut microbiota-short chain fatty acids-LPS-inflammatory axis. Reduction in liver fat accumulation and improvement of liver antioxidant ability.	[42]
	*Brassica oleracea* var. *italica* Plenck	The seeds were left to germinate for four days in the dark. Then, the seedlings were exposed to a 12 h photoperiod (light cycle) until they were harvested 10 days after they were sown. Microgreens were treated for two days with two different UVB levels and sprayed with 10 mM CaCl_2_.	Maintaining levels of health compounds during postharvest storage. The combination of UVB treatment and 10 mM CaCl_2_ prolonged overall quality and GSL levels until three weeks of storage.	[43]
Microgreens (continued)	*Brassica oleracea* var. *gemmifera* DC.	Microgreens were grown in commercial micro-farm and harvested without roots.	Prediction of nutritional outcome through in vitro simulated digestion and human fermentation ex vivo. *B. oleracea* var. *gemmifera* exhibited a high percentage of bio accessibility after simulated static gastrointestinal digestion and in vitro large intestine fermentation step for GSL/ITC.	[38]
	*Brassica oleracea* var. *gongylodes* L.	Microgreens were grown for 8–12 days after seeding at 21 °C under natural light conditions or in total darkness.	Chemoprevention of gastrointestinal cancer. GSL levels may be modulated by the length of sprouting time and light availability. The antinutritional progoitrin decreased after long time of sprouting, while darkness increased glucoerucin content. Microgreens contained lower levels of erucic acid when sprouting for 12 days compared to shorter periods of time of sprouting.	[44]
	*Raphanus sativus* L.	Microgreens were grown at standard conditions; drying trials were performed at different temperatures (40–60 °C).	Retention of bioactive compounds and nutritional quality for diverse dietary applications.	[45]
	*Raphanus sativus* L.	Microgreens were growth under varying Zn concentration and light intensity.	Modulation of nitrogen and energy metabolism for microgreens with an articulated regulation of GSL in response to Zn stress for improved functional-food potential.	[46]
	*Raphanus sativus* L.	Microgreens were primed using a commercial vermicompost product and cultivated in a vertical growth chamber under controlled light and temperature conditions.	Accumulation of GSL, and improvement in antioxidant activity.	[40]
	*Raphanus sativus* L.	Microgreens were grown in controlled conditions (22 °C temperature, 60% relative air humidity). Seeds were germinated for three days in darkness and then, until harvest (12 days), were grown under fluorescent or LED lamps with a photoperiod of 16 h light/8 h dark or continuous light.	Continuous LED light (red:green:blue ratio was 50.3:21.1:17.6) may improve antioxidant properties in microgreens.	[39]
Microgreens (continued)	*Sinapis alba* L.	Microgreens were cultivated in a vertical growth chamber under controlled light and temperature conditions.	Suppression of GSL and accumulation in polyphenols, and improvement in antioxidant activity.	[40]
	*Lepidium sativum* L.	Microgreens were cultivated in a vertical growth chamber under controlled light and temperature conditions.	Accumulation of polyphenols, and improvement in antioxidant activity.	[40]
	*Lepidium latifolium* L.	Microgreens were grown for 1, 2, 3 weeks in glass trays in an artificial climate chamber under controlled temperature (25° C), humidity (70%) and using a 12/12 h photoperiod. Trays were watered daily, and a half-strength basal MS medium was applied twice a week. Mature plants were separately grown in pots under the same conditions.	Accumulation of GSL and GSL hydrolysis products in the first weeks of growth in comparison with mature plants.	[47]
	*Lepidium latifolium* L.	Microgreens were growth in controlled conditions into an artificial climate chamber using a 12/12 h photoperiod at 25 °C. The trays were watered daily, and a half strength basal MS medium was applied twice a week. Microgreens were harvested at 1, 2, 3, 4, and 8 weeks, then they were immediately frozen in liquid nitrogen and freeze-dried.	Improvement of nutraceutical quality with reference to GSL and other phytochemical content and myrosinase activity. 3 weeks old microgreens are recommended as functional food as the richest source of sinigrin and benzyl GSL.	[48]
	*Brassica rapa* subsp. *nipposinica* (L.H.Bailey)	Microgreens were grown in peat moss substrate for 9 days (microgreens) or 29 days (baby greens) without fertilizers or pesticides.	Identification of 16 GSL, with 9 being up-regulated and 7 down-regulated during the development from microgreens to baby green stage. Network pharmacology identified 10 key targets and 24 bioactive compounds; probable activity against obesity and type 2 diabetes mellitus.	[49]
	*Brassica rapa* subsp. *nipposinica* (L.H.Bailey)	Microgreens were grown under standard light and temperature conditions, harvested after 12 days, freeze-dried and encapsulated using alginate.	The encapsulated sample was found to protect allyl ITC and sulforaphane during simulated gastric digestion and release them in the intestine for various dietary applications.	[50]
	*Brassica rapa* L.	Microgreens were grown in commercial micro-farm and harvested without roots.	Prediction of nutritional outcome through in vitro simulated digestion and human fermentation ex vivo. *B. rapa* exhibited a high percentage of bio accessibility after simulated static gastrointestinal digestion and in vitro large intestine fermentation step for anthocyanins and GSL/ITC.	[38]
Microgreens (continued)	*Brassica juncea* (L.) Czern	Microgreens were grown in commercial micro-farm and harvested without roots.	Prediction of nutritional outcome through in vitro simulated digestion and intestinal human fermentation ex vivo. *B. juncea* exhibited a high percentage of bioaccessibility after simulated static gastrointestinal digestion and in vitro large intestine fermentation step for lignans.	[38]
	*Brassica carinata* A. Braun	Microgreens were grown in three substrates (cocopeat, sand and a mix of cocopeat and sand) under four LED light spectra (Blue, Red, White and the mix of the three wavelengths) in a factorial experiment.	Safety of functional foods and nitrate reduction. White light and sand, or blue light and cocopeat seem to be the best combinations for achieving a reduction in nitrates; however, both solutions were associated with a reduction in carotenoids.	[51]
Sprouts (days from seeding to harvest 3–8)	*Brassica oleracea* var. *italica* Plenck	Sprouts were grown in microgravity and standard light/darkness conditions.	Enhancement of bioactive compound synthesis and antioxidant activity for diverse dietary applications.	[52]
	*Brassica oleracea* var. *italica* Plenck	Commercial sprouts were subjected to freeze-drying or air frying (160° for 10 min) thermal treatments.	Air frying enhanced phenolic acids, flavonols, sulforaphane, linolenic acid and phytosterol levels, α-Amylase inhibitory activity and maintained ACE inhibitory activity: air-frying represent a promising process for food product development.	[25]
	*Brassica oleracea* var. *italica* Plenck	Three cultivars of seeds were germinated in incubators under controlled conditions for 7 days.	Germination was associated with a decrease in total GSL and in enhancement of umami and sweet free amino acids, even if sensory analysis revealed bitterness and astringency as the predominant flavours. Control of germination process is needed to satisfy health benefits and consumer acceptance.	[53]
	*Brassica oleracea* var. *italica* Plenck	Disinfected seeds were soaked in a water bath at 30 °C for four hours, then they were spread in vermiculite and left to germinate in the dark for one day. Finally, they were placed in UV-A incubators at different intensities for five days.	Prevention and treatment of type II diabetes mellitus. UV-A irradiation at 12 W significantly induced health bioactive compound accumulation, including anthocyanins, polyphenols, ascorbic acid, GSL and sulforaphane in sprouts; furthermore, broccoli sprouts showed a significant inhibitory activity on α-amylase, α-glucosidase and pancrelipase.	[54]
Sprouts (Continued)	*Brassica oleracea* var. *italica* Plenck	Broccoli sprouts were cultivated in controlled conditions (25 °C and 80% relative humidity), receiving irrigation and treatments at different concentrations of CaCl_2_-HCl electrolyzed with a 3 A electrical current. Tap water was used as a control.	Improvement of antioxidant biomolecules and calcium in sprouts. The treatments at 5–15 mM CaCl_2_ progressively decreased epithiospecifier protein activity, while enhancing myrosinase activity, glucoraphanin and sulforaphane release.	[55]
	*Brassica oleracea* L.	Sprouts were grown in microgravity and standard light/darkness conditions.	Enhancement of bioactive compound synthesis and antioxidant activity for diverse dietary applications.	[52]
	*Brassica oleracea* var. *viridis* L.	Kale seeds were treated and sprouted in the presence of different treatments using selenium, sulfur or methyl jasmonate at three doses. The seeds were germinated in dark chambers at 25 °C and 85% relative humidity for 7 days.	Enhancement of bioactive compound synthesis and antioxidant activity for diverse dietary applications. 25 µM methyl jasmonate, 40 mg/L Selenium, 120 mg/L Sulfur treatments increased the accumulation of healthy GSL such as glucoraphanin and glucoerucin, while reduced unsafe GSL such as progoitrin in *Brassica oleracea*. Increases in lutein were also reported.	[56]
	*Brassica oleracea* var. *gongylodes* L.	Sprouts were grown in microgravity and standard light/darkness conditions.	Enhancement of bioactive compound synthesis and antioxidant activity for diverse dietary applications.	[52]
	*Brassica oleracea* var. *gemmifera* DC.	Sprouts were grown in microgravity and standard light/darkness conditions.	Enhancement of bioactive compound synthesis and antioxidant activity for diverse dietary applications.	[52]
	*Raphanus sativus* L.	Sprouts were growth in comparison to Broccoli sprouts for three days.	Hepatoprotective effect and upregulation of detoxifying enzymes in the liver, thanks to the high concentration of sulforaphene ITC.	[57]
	*Raphanus sativus* L.	Disinfected seeds were soaked in a water bath at 30 °C for four hours, then they were spread in vermiculite and left to germinate in the dark for one day. Finally, they were placed in UV-A incubators at different intensities for five days.	Prevention and treatment of type II diabetes mellitus. UV-A irradiation at 12 W significantly induced health bioactive compound accumulation, including anthocyanins, polyphenols, ascorbic acid, GSL and sulforaphene in sprouts; furthermore, a decrease in progoitrin was also observed.	[54]
	*Raphanus sativus* L.	Commercial sprouts, freeze dried and extracted in methanol at room temperature and further subjected to butyl alcohol extraction to remove free sugars. Extraction in ethanol, or 50% and 70% ethanol/Water were also characterized.	The sprouts of *R. sativus* may represent a functional food with high nutritional value and antioxidant activity. Of all the extracts, the 50% ethanol/water extract had the highest GSL content.	[58]
	*Moringa oleifera* Lam.	Sprouts were germinated at different temperatures (28–36 °C) and times (24–96 h) were evaluated.	Enrichment of nutrients, of bioactive molecules such as γ-aminobutyric acid (GABA), GSL, and antioxidant activity. Proteins, GABA, and GSL may accumulate under higher germination temperatures and longer germination times.	[59]

**Table 2:** The potential benefits of consuming Brassicales order species for the treatment of various diseases.

Disease	Brassicales Order Species	References
cardiovascular disease	*Diplotaxis tenuifolia* (L.) DC.	[22]
	*Brassica oleracea* L.	[60]
cardiovascular disease and diabetes mellitus	*Brassica oleracea* L.	[25]
	*Brassica oleracea* var. *capitata* L., *Brassica oleracea* var. *gemmifera* DC., *Brassica oleracea* var. *italica* Plenck	[54]
hyperthyroidism	*Nasturtium officinale* R.Br.	[24]
Parkinson’s disease	*Brassica oleracea* var. *capitata* L.	[26]
Alzheimer’s disease	*Brassica oleracea* var. *italica* Plenck	[27]
obesity-related disorders	*Brassica oleracea* var. *italica* Plenck	[31,42]
	*Moringa oleifera* Lam.	[36]
	*Brassica juncea* (L.) Czern	[33,34]
	*Brassica rapa* subsp. *nipposinica* (L.H.Bailey)	[49]
cancer	*Maerua subcordata* (Gilg) DeWolf	[61]
	*Raphanus sativus* var. *caudatus* (L.) Hook.f. & T.Anderson	[62]
	*Brassica oleracea* var. *gongylodes* L.	[44]
kidney disease	*Diplotaxis tenuifolia* (L.) DC., *Brassica oleracea* L.	[37]
liver disease	*Raphanus sativus* L.	[57]
	*Brassica oleracea* var. *viridis* L.	[32]
	*Brassica oleracea* var. *italica* Plenck	[42]

**Table 3:** The potential benefits of consuming fermented Brassicales order species for the treatment of various diseases.

Disease	Brassicales Order Species	References
intestinal diseases	*Brassica rapa* subsp. *pekinensis* (Lour.) Hanelt, *Raphanus sativus* L.	[66]
	*Brassica napus* L.	[67]
	*Brassica oleracea* var. *capitata* L.	[68]
cancer	*Carica papaya* L.	[69]
	*Brassica oleracea* var. *capitata* L.	[70]
	*Brassica oleracea* var. *italica* Plenck	[71]
autoimmune inflammatory disease	*Brassica oleracea* L.	[72]
airways pathologies	*Sisymbrium officinale* (L.) Scop.	[73]
foodborne pathogens	*Carica papaya* L.	[69]
antinutritional compounds in plant matrices	*Brassica oleracea* L. and *Lactiplantibacillus plantarum*	[74]

**Table 4:** Brassicales order by-products recently considered for nutraceutical applications.

Type of By-Product	Species	Nutraceutical Application	Ref.
Canola meals (fermented)	*Brassica napus* L.	Intestinal health and control of inflammation	[67]
Broccoli stalks (fermented)	*Brassica oleracea* var. *italica* Plenck	Functional foods with high antioxidant activity	[79]
Broccoli leaf		Neutralization of free radicals and regulation of postprandial blood glucose levels	[87]
IV-range broccoli residues (mainly stems)		Functional foods with high antioxidant activity	[86]
Broccoli florets and stems		Alzheimer’s disease	[85]
Broccoli stalks		Human intestinal inflammation	[84]
		Functional foods with high antioxidant activity	[83]
Broccoli leaves, stems, and florets		Weight loss applications	[82]
Non-marketable seeds, and industrial residues of packing and processing procedures		Glucoraphanin enriched ingredients	[80]
Non-marketable broccoli heads		GSL extraction	[81]
Leaf by-product	*Raphanus sativus* L.	obesity management	[88]
Leaf by-product	*Brassica oleracea* var. *capitata* L.	Functional foods with high antioxidant activity	[86]
Leaf by-product	*Brassica rapa* L.	Antibacterial and anticancer activities	[89]
Stems, roots, and peel	*Carica papaya* L.	Glucotropaeolin enriched ingredients and proteolytic activity in peel	[90]
Defatted seed meals	*Eruca vesicaria* subsp. sativa (Mill.) Hegi	Control of systemic markers of inflammation and lipid metabolism in adults	[91,92]
		Treatment of abdominal pain and diabetic neuropathic pain in animal models of induced neuropathy	[93,94]

**Table 5:** Main glucosinolates characteristic of plants of the Brassicales order with applications in functional nutrition.

Brassicales Order Species	Main Glucosinolates	Main Hydrolysis Product	Ref.
*Diplotaxis tenuifolia* (L.) DC.	(*R*_S_)-4-(Methylsulfinyl)butyl GSL (Glucoraphanin)	Sulforaphane	[20] [96]
	4-(Methylthio)butyl GSL (Glucoerucin)	Erucin	
	Dimeric 4-mercaptobutyl GSL	bis(4-isothiocyanatobutyl) disulfide	
*Diplotaxis muralis* DC.	(*R*_S_)-4-(Methylsulfinyl)butyl GSL (Glucoraphanin)	Sulforaphane	[23]
	4-(Methylthio)butyl GSL (Glucoerucin)	Erucin	
*Eruca vesicaria* subsp. *sativa* (Mill.) Hegi	(*R*_S_)-4-(Methylsulfinyl)butyl GSL (Glucoraphanin)	Sulforaphane	[97]
	Dimeric 4-mercaptobutyl GSL	bis(4-isothiocyanatobutyl) disulfide	
*Nasturtium officinale* R.Br.	2-Phenylethyl GSL (Gluconasturtiin)	Nasturtiin	[24] [98]
	4-(Methylthio)butyl GSL (Glucoerucin)	Erucin	
	Benzyl GSL (Glucotropaeolin)	Benzyl ITC	
	(*R*_S_)-3-(Methylsulfinyl)propyl GSL (Glucoiberin)	Iberin	
*Brassica oleracea* L.	Indol-3-ylmethyl GSL (Glucobrassicin)	Indole-3-carbinol	[99]
	Allyl GSL (Sinigrin)	Allyl ITC	
*Brassica oleracea* var. *capitata* L.	Indol-3-ylmethyl GSL (Glucobrassicin)	Indole-3-carbinol	[70] [41]
	Allyl GSL (Sinigrin)	Allyl ITC	
	(*R*_S_)-3-(Methylsulfinyl)propyl GSL (Glucoiberin)	Iberin	
*Brassica Oleracea* var. *botrytis* L.	Indol-3-ylmethyl GSL (glucobrassicin)	Indole-3-carbinol	[100]
	2-Phenylethyl GSL (Gluconasturtiin)	Nasturtiin	
*Brassica oleracea* var. *gemmifera* DC.	Indol-3-ylmethyl GSL (Glucobrassicin)	Indole-3-carbinol	[101]
	Allyl GSL (Sinigrin)	Allyl ITC	
	(2*R*)-2-Hydroxybut-3-enyl GSL (Progoitrin)	(S)-5-vinyl-1,3-oxazolidine-2-thione (goitrin)	
*Brassica oleracea* var. *gongylodes* L.	4-(Methylthio)butyl GSL (Glucoerucin)	Erucin	[44]
	(*R*_S_)-3-(Methylsulfinyl)propyl GSL (Glucoiberin)	Iberin	
*Brassica oleracea* var. *italica* Plenck	(*R*_S_)-4-(Methylsulfinyl)butyl GSL (Glucoraphanin)	Sulforaphane	[31]
	Indol-3-ylmethyl GSL (Glucobrassicin)	Indole-3-carbinol	
*Brassica oleracea* var. *viridis* L.	(*R*_S_)-3-(Methylsulfinyl)propyl GSL (Glucoiberin)	Iberin	[32]
	Indol-3-ylmethyl GSL (Glucobrassicin)	Indole-3-carbinol	
	(*R*_S_)-4-(Methylsulfinyl)butyl GSL (Glucoraphanin)	Sulforaphane	
*Brassica juncea* (L.) Czern	Allyl GSL (Sinigrin)	Allyl ITC	[33] [34]
*Brassica rapa* subsp. *chinensis* (L.) Hanelt	But-3-enyl GSL (Gluconapin)	Napin	[35]
	Pent-4-enyl GSL (Glucobrassicanapin)	4-Pentenyl ITC	
	(2*R*)-2-Hydroxybut-3-enyl GSL (Progoitrin)	(S)-5-vinyl-1,3-oxazolidine-2-thione (goitrin)	
*Moringa oleifera* Lam	4′-O-acetyl-4-(α-l-rhamnopyranosyloxy)benzyl GSL (acetylated Isomer III Glucomoringin)	4-acetyl moringin (III)	[14]
	4-(α-l-rhamnopyranosyloxy) benzyl GSL (Glucomoringin)	moringin	
*Raphanus sativus* L.	(3*E*)-4-(Methylsulfanyl)but-3-enyl GSL (Glucoraphasatin or Dehydroglucoerucin)	4-(Methylsulfanyl)but-3-enyl ITC ^a^	[102]
	(*R*_S_, 3*E*)-4-(Methylsulfinyl)but-3-enyl GSL (Glucoraphenin)	Sulforaphene	
*Sinapis alba* L. (seeds)	4-Hydroxybenzyl GSL (Sinalbin)	4-Hydroxybenzyl ITC ^b^	[103]
*Lepidium sativum* L. (microgreens and seeds)	Benzyl GSL (Glucotropaeolin)	Benzyl ITC, Benzyl cyanide ^c^	[104]
*Lepidium latifolium* L. (microgreens)	Allyl GSL (Sinigrin)	1-cyano-2,3-epithiopropane Allyl ITC	[47] [48]
	Benzyl GSL (Glucotropaeolin)	Benzyl ITC	
*Brassica rapa* subsp. *nipposinica* (L.H.Bailey) (microgreens)	Allyl GSL (Sinigrin)	Allyl ITC	[50]
	(*R*_S_)-3-(Methylsulfinyl)propyl GSL (Glucoiberin)	Iberin	
	(*R*_S_)-4-(Methylsulfinyl)butyl GSL (Glucoraphanin)	Sulforaphane	
*Brassica rapa* L. (microgreens)	4-(Methylthio)butyl GSL (Glucoerucin)	Erucin	[38] [105]
	Pent-4-enyl GSL (Glucobrassicanapin)	4-Pentenyl ITC	
	(*R*_S_)-4-(Methylsulfinyl)butyl GSL (Glucoraphanin)	Sulforaphane	
*Brassica carinata* A. Braun	Allyl GSL (Sinigrin)	Allyl ITC	[51]
*Sisymbrium officinale* (L.) Scop.	Isopropyl GSL (Glucoputranjivin)	Iso-propyl ITC	[73] [106]
*Carica papaya* L.	Benzyl GSL (Glucotropaeolin)	Benzyl ITC	[90]

^a^ 4-(Methylsulfanyl)but-3-enyl ITC undergoes spontaneous cyclization at room temperature and is highly unstable in aqueous environments [107]. ^b^ *para* hydroxylation of 4-hydroxybenzyl ITC results in hydrolysis to benzylic alcohols and thiocyanate ion in aqueous solutions [108,109]. ^c^ Benzyl thiocyanate, Benzyl alcohol, and Benzaldehyde have also been identified in autolysates of seeds and young plants of *L. sativum* [104,110].

## Data Availability

No new data were created or analyzed in this study. Data sharing is not applicable to this article.

## Abbreviations

The following abbreviations are used in this manuscript:

ACE	Angiotensin converting enzyme
GSL	Glucosinolate
ITC	Isothiocyanate

## Appendix A

Table A1 provides a comprehensive overview of fermented foods derived from species belonging to the Brassicales order which have attracted considerable attention in recent years due to their beneficial health properties.

**Table A1:** Fermented foods or products obtained from Brassicales order species.

Brassicales Order Species	Processing	Nutraceutical Application	Ref.
*Brassica rapa* subsp. *pekinensis* (Lour.) Hanelt	Kimchi samples from conventional markets in Daegu, South Korea.	Integrated analysis of bioactive molecules and metagenomic characteristics of Korean fermented foods which may support gut health and exhibit antioxidant, anti-inflammatory, anti-cancer, antiviral, antidiabetic, antihypertensive, and anti-obesity properties.	[66]
*Raphanus sativus* L.	Kimchi samples from conventional markets in Daegu, South Korea.	See description above.	[66]
*Brassica napus* L.	Commercial canola meals was fermented with *Bacillus licheniformis* DY145.	Functional fermented foods aimed at promoting gut health, *Lactobacillus* proliferation, and reduced inflammation.	[67]
*Carica papaya* L.	The papaya pulp, having been deseeded and skinless, underwent a fermentation process with the following strains: *Enterobacter xiangfangensis*, *Lactiplantibacillus plantarum*, *Limosilactobacillus fermentum*, *Lactococcus hircilactis*, *Lactococcus lactis*, *Lactococcus lactis* subsp. *Lactis*, *Levilactobacillus brevis*, and *Pedicoccus pentosaceus* to produce a water kefir.	Antibacterial activity against foodborne pathogens (*Escherichia coli*, *Staphylococcus aureus*, *Bacillus cereus*, and *Salmonella* sp.); chemopreventive potential assessed in cancer cell lines. Mild alcohol content (1–3%).	[69]
*Brassica oleracea* var. *capitata* L.	Pieces of cabbage were pre-inoculated with non-pathogenic *Escherichia coli* ATCC 8739, and natural microbiota in fresh cabbage, and then divided into groups for UV, ultrasound and combined UV/ultrasound treatments. The fermentation was activated on shredded cabbage pretreated with UV or ultrasound adding *Leuconostoc mesenteroides* (KTCT3505) as starter culture.	Fermentation approach led to a significant increase in S-methylmethionine content, known as a molecule able to alleviate dyspeptic symptoms in patients with chronic gastritis, and to a significant increase in the amino acid content.	[68]
*Brassica oleracea* var. *capitata* L.	Commercial white cabbage was shredded and mixed with carrots and 3% (*w*/*w*) sodium chloride before spontaneous fermentation. The fermentation process was monitored for 17 weeks, with the jars kept in the dark at 5 °C.	GSL and their corresponding breakdown products were characterized both in white cabbage and sauerkraut juice, considered as a by-product of fermentation. The study evidenced that both sauerkraut and its juice are a good source of ascorbigen and ITCs, and that the juice, starting from 2 weeks of fermentation, can be considered a functional food with anticancer properties.	[70]
*Brassica oleracea* var. *capitata* L.	One strain of *Enterobacter xiangfangensis* isolated from fermented Thai fish and one strain of *Enterococcus casseliflavus* isolated from fermented Thai cabbage were tested for high myrosinase activity. The strains were used as starter cultures at 10^6^ CFU/mL in cabbage head-rice water fermentation (25 °C for three days).	Myrosinase-positive bacteria, used as starter cultures, may enhance the production of effective ITCs such as sulforaphane and iberin, which have several health benefits.	[75]
*Brassica oleracea* L.	Raw and blanched sprouts were evaluated after spontaneous and *Lactiplantibacillus plantarum*-inoculated fermentation.	Safe functional food with minimum content of phytates, tannins, and oxalates. Blanched, *L. plantarum* inoculated fermentation showed the best performances.	[74]
*Brassica oleracea* L.	Fresh leaves of three cultivar were washed with tap water, air dried for 4 h at room temperature and subjected to fermentation with a starter culture (*Lactiplantibacillus* (Lpb.) *plantarum* 332, *Lpb. paraplantarum* G2114, and *Pediococcus pentosaceus* 2211, or spontaneous fermentation, for 14 days at room temperature.	Increase in bioactive molecules bioavailability and of antioxidant, cyto-protective properties and immunomodulatory activities.	[72]
*Brassica oleracea* L.	Fresh cabbage was cut and added to two low salt concentrations solution (0.5% or 1%) and inoculated with *Lactobacillus plantarum*, *Leuconostoc mesenteroides* or a combination, at room temperature for 5 days. Spontaneous fermentation in 2% salt solution was used as a control.	Enrichment of antioxidant activity and ITC content. Sauerkraut obtained at low salt concentrations thanks to *L. mesenteroides* culture exhibited the highest antioxidant activity and a higher content of sulforaphane in comparison to the control.	[113]
*Brassica oleracea* var. *italica* Plenck	Commercial broccoli heads were cut, sanitized, and ground to obtain homogeneous pieces of 2–4 mm thick. Spontaneous and induced fermentation with *Levilactobacillus brevis* (3M1) and *Lactococcus lactis* (3M8) strains as starter culture, were performed at 23° C for 10 days. Samples were stored at −80 °C and freeze-dried for final analysis.	Functional foods with protective effects against induced oxidative effects and antiproliferative and anti-inflammatory activities in colon cancer cells. The highest GSL content and antioxidant activity were detected in day 6 of fermentation (both spontaneous and induced).	[71]
*Brassica oleracea* var. *italica* Plenck	Broccoli stalks were cut into 6 mm slices and jar packed adding brine (6% *w*/*v*). Anaerobic, spontaneous fermentation was carried out at 25 °C in dark conditions for six days in the presence of garlic gloves (8 g/80 g broccoli stalks) or mustard seeds (10 g/80 gbroccoli stalks) as dressings. The samples were stored at 4 °C for further six days.	Functional fermented food with antioxidant activity and high content of phenolic compounds. GSLs were tested and decreased during fermentation, probably enriching covering liquid in ITCs, thanks to mustard seed dressing. The determination of these compounds is lacking but discussed in the manuscript.	[79]
*Brassica oleracea* var. *italica* Plenck	1:1 *Lactobacillus plantarum* and *Leuconostoc mesenteroides* strains, previously isolated from broccoli, were used as a mixed starter culture for broccoli puree fermentation. Broccoli florets were homogenized with water in a ratio 3:2 and inoculated with 8 log CFU/g. Fermentation was carried out at 30 °C until pH 4 (4 days of incubation). Then one lot was stored at 4 °C, and a second lot at 25 °C.	Improving yield and stability of sulforaphane in a functional ingredient.	[77]
*Brassica oleracea* var. *italica* Plenck	Broccoli florets puree was prepared in ice-cold water with a ratio 1:1 broccoli/water and 1 min of homogenization, then it was immediately packed and autoclaved at 121 °C for 3 min. Broccoli puree was inoculated with 10% (*v*/*v*) suspension of lactic acid bacteria isolated from leaves (5 *Lactobacillus plantarum* strains) and florets of Broccoli (2 *Leuconostoc mesenteroides* strains). Fermentation was achieved after 15 h at 30 °C.	Enhancing bioactive molecules for the functional food industry. Fermentation increased GSL and glucoraphanin levels in broccoli puree with two strains of ferments, that is, the *L. plantarum* and *L. mesenteroides* with high capacity for glucose and fructose utilization.	[76]
*Moringa oleifera* Lam.	Leaves were pretreated with water at 90 °C to eliminate natural bacteria and after cooling were inoculated with ten different lactobacilli strains.	*Pediococcus acidilactici*, *P. pentosaceus*, *L. Lactiplantibacillus plantarum*, *Levilactobacillus brevis*, and *Leuconostoc mesenteroides* exhibited notable growth and viability in moringa leaves during the fermentation process, with *P. acidilactici* CECT 98, *L. plantarum* CECT 9567, and *L. mesenteroides* CECT 219 T able to increase antioxidant activities and change quali-quantitative profile in phenolic compounds.	[114]
*Sisymbrium officinale* (L.) Scop.	Artisanal beer: starting from a malt extract, *S. officinalis* (1 g/L) was added as commercial dried aerial parts (60 °C for 5 min). The solution (6 L) was inoculated with 5.5 g yeast and fermentation was kept at 20 °C for 7 days. Then sugar was added, and beer was bottled and stocked for 2 weeks at room temperature. Kombucha: 5 g of *S. officinalis* flowers and 40 g of sucrose were added in 1 L boiling water. The solution was left to infuse for 20 min and, after cooling and filtering, was transferred into sterile glass vessel and SCOBY was added. The jar was covered with a sterile gauze and left to ferment at room temperature for 12 days.	Treatment of respiratory discomfort, and airways pathologies. Kombucha had undetectable content of ITCs, while *S. officinalis* enriched beer had about 8 µg active ITC per portion.	[73]
